# Minimally invasive resection of heart valve papillary fibroelastoma: two case reports and review of the literature

**DOI:** 10.1186/s13019-023-02392-1

**Published:** 2023-11-13

**Authors:** Thuan Q. Phan, Chuong T. V. Pham, Vinh D. A. Bui, Thang D. Ho, Thao N. Le, Thanh V.T. Nguyen, Dang Nguyen, Minh N. Vuong, Dinh H. Nguyen

**Affiliations:** 1 Department of Adult Cardiovascular Surgery, University Medical Center HCMC, University of Medicine and Pharmacy at Ho Chi Minh City, Ho Chi Minh City, Vietnam; 2 Department of Cardiovascular and Thoracic Surgery, Faculty of Medicine, University of Medicine and Pharmacy at Ho Chi Minh City, Chi Minh City, Vietnam; 3 Massachusetts General Hospital, Corrigan Minehan Heart Center, Harvard Medical School, Boston, MA USA

**Keywords:** Papillary fibroelastomas, Beating heart total thoracoscopy, Upper hemi-sternotomy

## Abstract

**Background:**

Cardiac papillary fibroelastomas are rare, accounting for approximately 10% of all cardiac tumors, with 44% of cases located on the aortic valve and only 15% of cases located on the tricuspid valve. However, the optimal management of papillary fibroelastomas remains varied.

**Case presentation:**

We present two successful instances of treating heart valve papillary fibroelastomas through minimally invasive surgery. These cases involved heart valve papillary fibroelastomas located in two common sites: the aortic valve on the left heart, which was accessed via an upper hemi-sternotomy, and the tricuspid valve on the right heart, which was accessed via beating heart total thoracoscopy.

**Conclusion:**

The article consistently demonstrates the effectiveness of a minimally invasive surgical approach in managing heart valve papillary fibroelastomas. This study provides further evidence by presenting two cases of heart valve papillary fibroelastomas - one on the aortic valve and the other on the tricuspid valve - that were successfully treated using this approach, resulting in favorable outcomes.

## Background

Papillary fibroelastomas (PFE) are primary tumors of cardiac origin [1]. The term ‘papillary fibroelastoma’ was first introduced by Cheitlin in a 1975 case report, which described an incidental discovery during autopsy [2]. Papillary fibroelastoma accounts for approximately 10% of all cardiac tumors and may be the most common type of primary cardiac tumor [1, 2]. They are most commonly found on heart valves, with a prevalence rate of over 80%, and the aortic valve is the most common site of occurrence, accounting for 44% of case [2–7]. Nevertheless, PFE can also occur in the right cardiac chamber, with the tricuspid valve being the primary location, observed in around 15% of cases [8, 9]. The etiology of PFE is unclear. Recent hypotheses propose that the pathogenesis of PFE may involve fibroblast infiltration with organization of mural thrombi, viral-induced tumor growth, and an endothelial response to cardiac surgery, mechanical trauma, or thoracic radiation [1].

Papillary fibroelastomas predominantly affect males and individuals over the age of 40, although they can occur at any age [2, 10]. The clinical manifestation of PFE displays a wide range of variability, with approximately 54% of cases being asymptomatic at the time of diagnosis [2]. However, symptomatic events such as extraction, limb embolism, coronary artery or embolism can lead the patient to seek exploration, as they may indicate the underlying obstruction causing the symptoms [1]. Vegetations are among the differential diagnoses for PFE and can occasionally be misdiagnosed and inappropriately treated with antibiotics [11].

The definitive diagnosis of PFE relies on histopathology, but initial diagnosis is frequently established via imaging modalities, particularly transthoracic echocardiography (TTE), which has an accuracy rate of approximately 85%. PFE that are smaller than 5 mm often require transesophageal echocardiography (TEE) for evaluation. Therefore, when PFE are suspected and thoracic echocardiography results are negative, transesophageal echocardiography should be recommended [2]. Additionally, cardiac computed tomography (CCT) and cardiac magnetic resonance (CMR) serve as secondary imaging modalities in the diagnosis of PFE [8]. PFE has been noted to exhibit a hypo-intense signal on cine images and an intermediate signal intensity on both T1 and T2 weighted images, resembling that of myocardial tissue. When it comes to cardiac CT imaging, PFE presents as a mass with hypodense characteristics and irregular borders [2]. Moreover, CMR and CCT are highly valued not only for their ability to visualize coronary anatomy and correlate tumor location with adjacent cardiac structures, but also for developing a less invasive treatment strategy. These imaging modalities are also capable of distinguishing PFE from other cardiac pathologies such as vegetations and thrombosis. The differential diagnoses for PFE are extensive and include valvular vegetation, Lambl’s excrescences, myxoma, cysts, thrombus, fenestration, fibroma, and artifacts. PFE located outside of the valves can pose an even more complex diagnostic challenge. Tamin et al. recommends that in such cases, blood cultures, antiphospholipid antibody testing, and screening for systemic lupus erythematosus should be performed to aid in the diagnostic process [2].

Papillary fibroelastomas exhibit a macroscopic appearance characterized by numerous frond-like fibrous projections that give rise to a “sea anemone” appearance when immersed in saline. The histological features of the tumor consist of an avascular fibroelastic core composed of a hyalinized collagen matrix, with a rim of smooth muscle cells and elastic fibers, and lined by endocardial endothelium [1, 2].

The current medical literature lacks clear, standardized protocols for the management of PFE. Despite PFE’s benign histology, however, given the potential for severe embolic complications, surgical intervention is strongly advised following the diagnosis of these tumors. In order to achieve optimal outcomes, meticulous care must be taken during the operative procedure to ensure the complete removal of the tumor, including the stalk and underlying endocardium, while avoiding the possibility of fragmentation, embolization, or the presence of residual tumor [1, 8]. Surgical excision remains the preferred treatment modality for symptomatic patients who meet the criteria for surgery, as defined by a Society of Thoracic Surgeons score of less than 1% [2]. For symptomatic patients who are not considered to be surgical candidates, long-term antiplatelet or anticoagulation therapy may be initiated [1, 2]. Asymptomatic patients should be evaluated for surgical excision if the tumor size is greater than 1 cm or if there is evidence of increased mobility, as these factors are associated with a higher risk of nonfatal embolization or death. Asymptomatic patients with small (< 1 cm) and non-mobile (i.e. lacking a stalk) left-sided tumors may be managed conservatively through clinical observation, without immediate intervention [2].

While median sternotomy remains the primary surgical approach, recent evidence suggests that minimally invasive techniques can offer favorable exposure and safe resection, particularly for aortic or tricuspid valve papillary fibroelastomas (PFE), in the absence of co-existing pathologies requiring open cardiac surgery [1, 2, 6, 8, 12–15]. Minimally invasive surgery presents potential advantages, including improved cosmetic outcomes, reduced post-operative pain, decreased blood loss and transfusion requirements, as well as shorter stays in the intensive care unit and overall hospitalization durations [1]. Nonetheless, it is worth noting that the adoption of minimally invasive surgery may be hindered by a steep learning curve for operators and the need for costly equipment, thereby limiting its uptake in smaller medical centers. Additionally, there remains a dearth of data regarding the optimal surgical approach for PFE, and the rarity of these tumors makes it improbable that a randomized controlled trial will be conducted in the foreseeable future [1, 8, 12].

This article offers a thorough review of the literature regarding minimally invasive surgical excision techniques for the management of PFE, and presents two successful cases of PFE treatment utilizing these methods. Specifically, the cases involved PFE located in two common sites, namely the left heart on the aortic valve accessed via an upper hemi-sternotomy, and the right heart on the tricuspid valve via beating heart total thoracoscopy.

## Case presentation

### Case 1

**Fig. 1:**
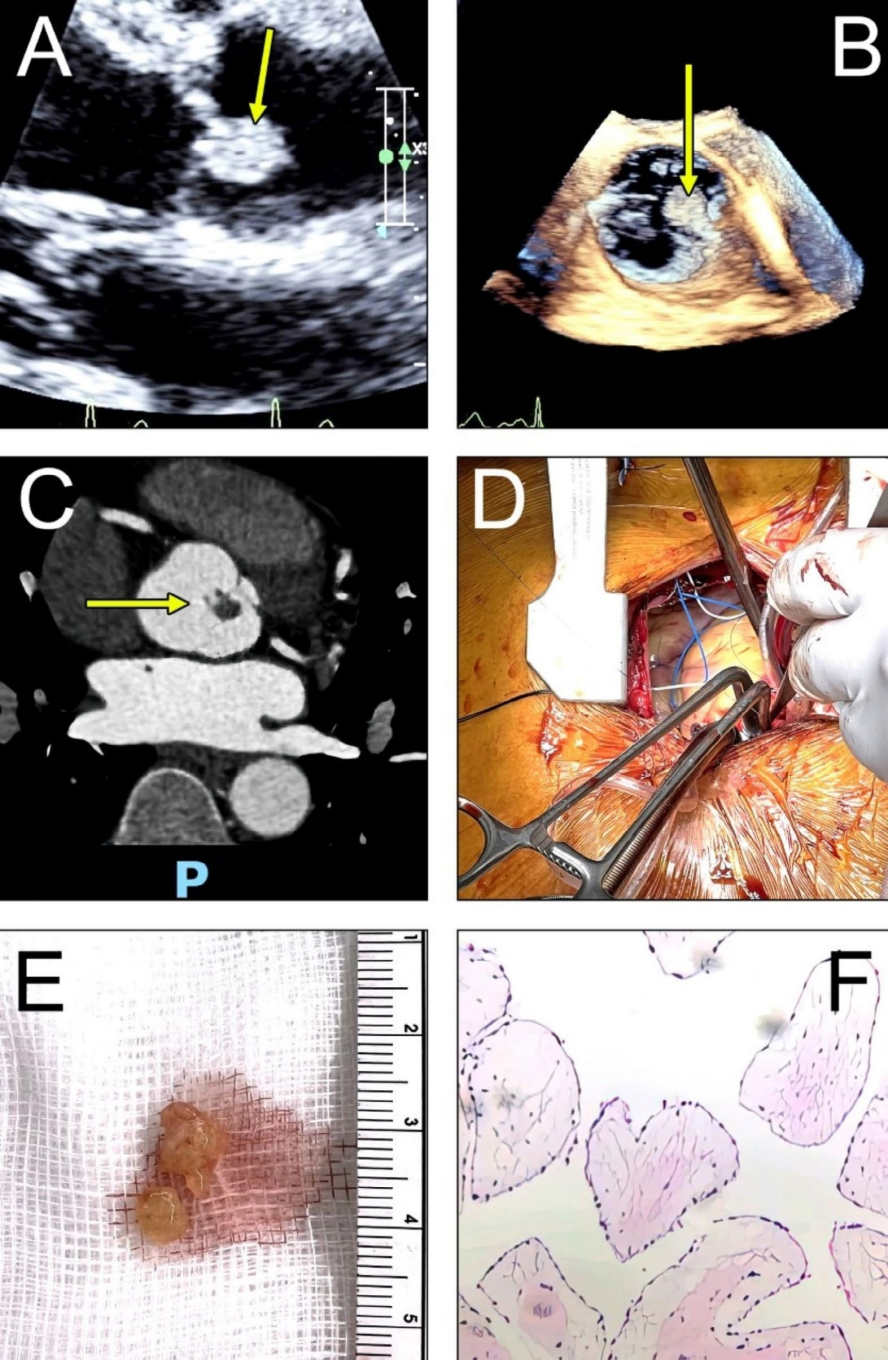
**A, B**: TTE/ Three-dimensional TEE images of PFE on the right coronary cusp of the aortic valve, **C**: CCT shows PFE on the right coronary cusp of the aortic valve ,**D**: Minimal access approach and set-up, E: Fibroelastoma that has been removed, F: The histological section of the tumor depicts a central core of hyalinized connective tissue that lacks blood vessels, enveloped by a connective matrix and enclosed by a solitary layer of endocardial cells. (The arrow denotes the location of the tumor)

A 51-year-old man visited the hospital clinic due to experiencing shortness of breath during physical exertion. He had a personal history of smoking for 10 pack-years and no significant co-morbidities. Despite a normal electrocardiogram, an TTE revealed the presence of a tumor on the aortic valve (Fig. 1A), leading to an emergency admission to the hospital’s emergency department.

Upon admission, a thorough physical examination did not uncover any significant abnormalities. Electrocardiography showed normal sinus rhythm, and all laboratory blood results were unremarkable. TEE revealed a normal left ventricular ejection fraction, with the remaining heart valves showing no notable abnormalities. However, a mass displaying echogenic properties and measuring 6.9 × 9.5 mm has been detected on the right coronary cusp of the aortic valve, indicating a high mobility PFE. Owing to its characteristics, this discovery presents a substantial risk of embolism (Fig. 1B). Following a CCT examination, the patient was found to possess a normal coronary artery and a tumor located on the right coronary cusp of the aortic valve, as well as a mixed echogenic myocardial cross-density measuring 10 × 8 mm, with the suspicion of PFE (Fig. 1C). The Society of Thoracic Surgeons score for the patient is 0.91%.

During the surgical procedure, the patient underwent a minimally invasive approach involving a 6 cm incision to access the heart through a limited upper sternotomy that extended to the left fourth intercostal space. Cannulation of the ascending aorta and right femoral vein was performed (Fig. 1D), followed by transverse aortotomy for inspection. A round mass located at the edge of the right coronary cusp was identified and excised while preserving the integrity of the aortic valve. The lesion was excised while preserving the aortic valve, and its perfect competence was confirmed before closure. The total cross-clamp time was 34 min, with a bypass time of 60 min. Intraoperative TEE confirmed normal valve function and good myocardial function before decannulation. The excised lesion measured 9 × 10 × 2 mm (Fig. 1E), and histopathological analysis showed a papillary lesion consisting of cores of collagen surrounded by elastic tissue covered with a layer of endocardial endothelium, which is typical of PFE (Fig F). The patient was extubated 5 h after the surgery and was discharged home four days later without any complications.

### Case 2

**Fig. 2:**
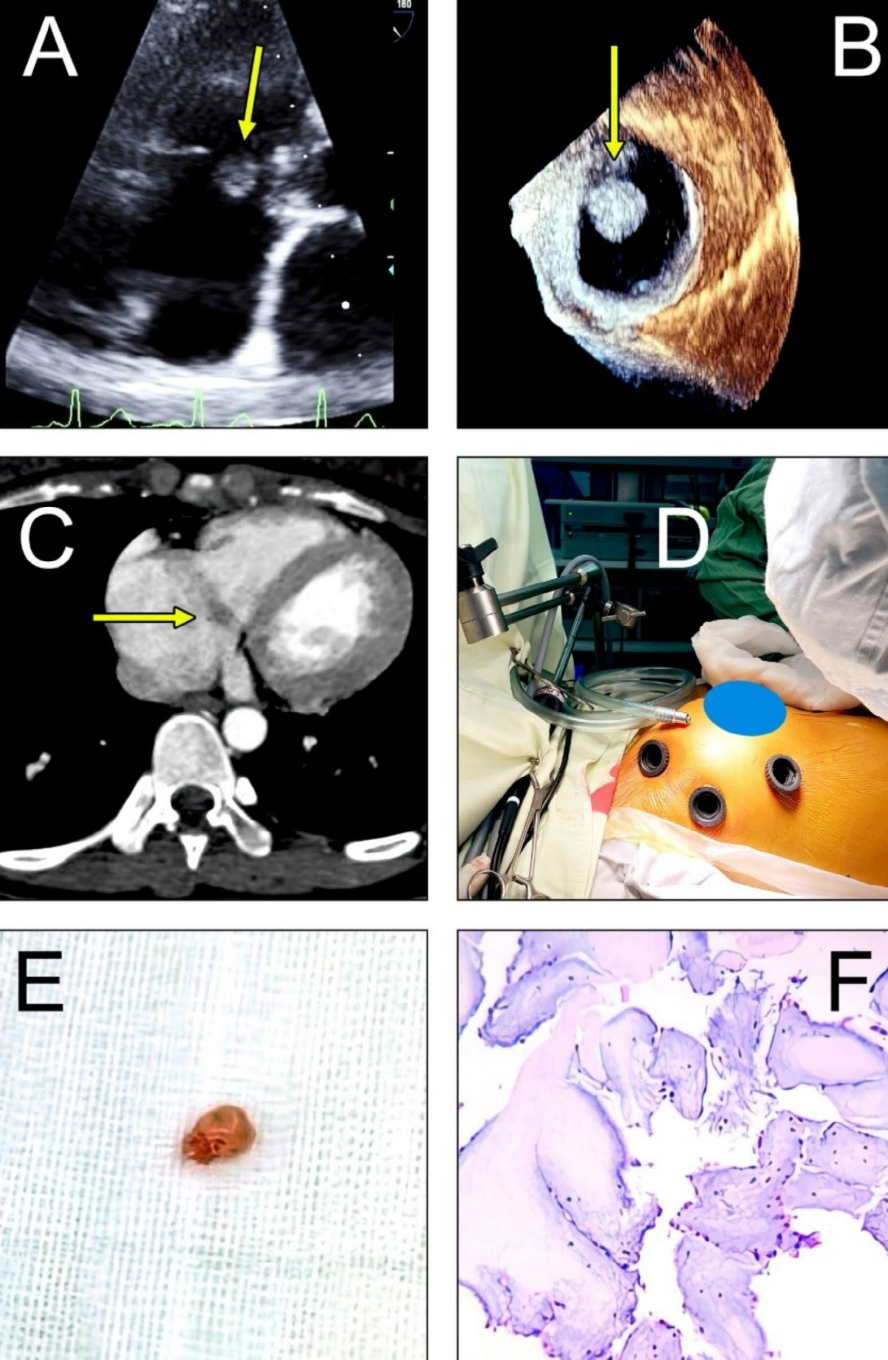
**A, B**: TTE/ Three-dimensional TEE images of PFE on the septal leaflet of the tricuspid valve, **C**: CCT shows PFE on the septal leaflet of the tricuspid valve, **D**: Minimal access approach and set-up, **E**: Fibroelastoma that has been removed, F: The histological section of the tumor depicts a central core of hyalinized connective tissue that lacks blood vessels, enveloped by a connective matrix and enclosed by a solitary layer of endocardial cells. (The arrow denotes the location of the tumor)

A 17-year-old female, with no significant co-morbidities and no apparent cardiovascular risk factors, presented to the hospital clinic due to exertional dyspnea. Despite having a normal electrocardiogram and chest X-ray, a TTE detected a tumor on the tricuspid valve (Fig. 2A), which prompted an emergency admission to the hospital’s emergency department.

Upon admission to the hospital, the patient underwent a comprehensive physical examination which revealed no significant abnormalities. The electrocardiogram showed normal sinus rhythm and all laboratory blood results were within the normal range. TEE revealed a normal left ventricular ejection fraction and no notable abnormalities in the remaining heart valves. However, a highly mobile PFE was detected on the septal leaflet of the tricuspid valve, measuring 8.7 × 9.6 mm and exhibiting echogenic properties. Due to its characteristics, this finding posed a significant risk of embolism (Fig. 2B). Further CCT imaging confirmed a normal coronary artery and confirmed the presence of a tumor on the septal leaflet of the tricuspid valve. In addition, a mixed echogenic cross-density of the myocardium measuring 10 × 7.2 mm was identified, raising suspicion of PFE (Fig. 2C).

The patient underwent non-robotic totally thoracoscopic surgery using three trocars. One trocar was placed in the 2nd intercostal space and midclavicular line, another in the 4th intercostal space and anterior axillary line, and the last one in the 5th intercostal space and midclavicular line. Cannulation of the artery and right femoral vein was performed (Fig. 2D), followed by the opening of the right atrium, revealing a round mass located at the septal leaflet of the tricuspid valve. The lesion was excised while preserving the tricuspid valve, and its perfect function was confirmed before closure. The procedure required a 69-minute bypass time, and the procedure was performed on a beating heart. Intraoperative TEE confirmed normal valve function and good myocardial function prior to decannulation. The excised lesion measured 10 × 8 × 2 mm (Fig. 2E), and histopathological analysis showed a papillary lesion consisting of cores of collagen surrounded by elastic tissue covered with a layer of endocardial endothelium, which is typical of PFE (Fig. 2F). The patient was extubated six hours after the surgery and discharged home three days later without any complications.

**Fig. 3:**
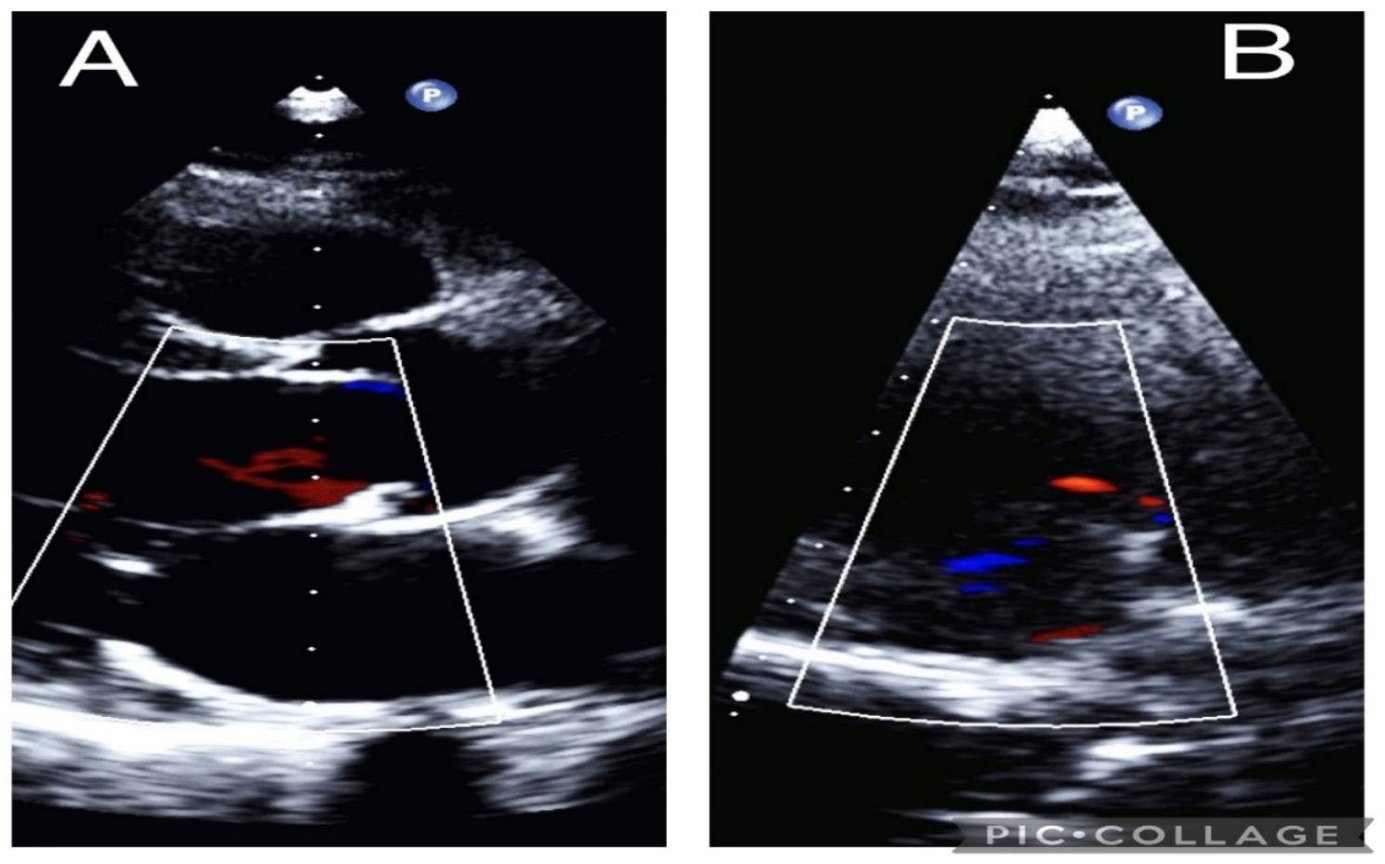
The echocardiogram conducted 6 months after the surgery revealed no indications of new tumor growth (**A**: Case 1, **B**: Case 2)

Both patients were monitored for a period of six months, during which they underwent echocardiographic evaluation that showed no evidence of new tumor growth, and their heart valves were found to be functioning within the normal range (Fig. 3A and B).

## Discussion and conclusions

Patients with PFE frequently seek medical attention due to the presence of symptoms that are not directly attributable to tumor-related complications [1]. Exertional dyspnea is a commonly observed atypical manifestation [8, 16]. In our case, the patients presented with non-specific clinical symptoms, which were not closely associated with PFE, including exertional dyspnea. However, these symptoms prompted the performance of an echocardiogram, leading to the detection of the tumor. Prompt intervention was initiated to mitigate the risk of potential complications. Subsequently, both patients remained asymptomatic during their follow-up after discharge.

Considering the non-specific clinical presentation of patients with PFE, diagnostic imaging has become the foremost approach for the detection of this condition. Transthoracic echocardiography (TTE) remains a dependable and economical diagnostic modality, exhibiting a sensitivity and specificity of nearly 90% for detecting tumors with a size greater than 0.2 cm [8]. Transesophageal echocardiography (TEE) represents a valuable tool in the confirmation of PFE, particularly for tumors that may be small or valuable for evaluation of motility and pedicle of tumor. In both cases, our patients underwent transesophageal echocardiography to confirm the diagnosis and decision to surgical intervention.

Although TTE and TEE are diagnostic modalities that can effectively identify PFE, CCT remains a valuable tool for detecting potential complications and associated lesions, as well as for performing preoperative coronary evaluations [8]. In both of our cases, CCT was performed to evaluate associated lesions and assess the coronary arteries. PFE tumors are characterized by a macroscopic appearance featuring numerous frond-like fibrous projections that create a distinctive “sea anemone” appearance when immersed in saline solution [2]. Consequently, the CCT images of our two patients reveal that the tumor exhibits heterogeneity, which may be attributed to the infiltration of contrast into the tumor fibers. This represents the highlighted image on CCT of PFE [2].

The presence of high mobility and risk of embolism in PFE situated on the aortic valve signify an urgent requirement for surgical intervention. The removal of PFE from the aortic valve is commonly achieved through the use of minimally invasive approaches. Among these approaches, the upper hemi-sternotomy method is widely employed and considered effective [1]. Additionally, the Right Anterior Thoracotomy Approach has been reported as a successful technique for excising PFE located on the aortic valve [13]. Furthermore, some institutions employ completely endoscopic techniques, whether robotic or non-robotic, to achieve PFE removal from the aortic valve [12, 17]. In our case, two factors influenced our choice of the semi-sternal thoracotomy approach: firstly, robotic surgery was not available at our institution; and secondly, the patient’s ascending aortic anatomy was significantly leftward, making the semi-sternal thoracotomy a more appropriate option. Given these considerations, we opted for this minimally invasive technique, resulting in a relatively short aortic clamping time of 34 min and an cardiopulmonary bypass (CPP) time of 60 min, which is comparable to the findings reported by other authors.

Despite its rarity, the existence of PFE on the tricuspid valve mandates surgical intervention under specific conditions, including heightened mobility, elevated susceptibility to pulmonary embolism, partial impingement of the right ventricular outflow tract, or plausible correlation with cardiac rhythm disturbances [8]. In cases where tumors are located in this anatomical site, the conventional approach of median sternotomy with CPP is typically employed for tumor excision [18]. Alternatively, an assisted thoracoscopic technique through right thoracotomy with CPP may be considered for tumor removal. The utilization of a minimally invasive approach for the complete thoracoscopic removal of tumors with beating heart can serve as a feasible and efficacious alternative for treating such tumors, provided that the patient does not exhibit other cardiovascular comorbidities that necessitate simultaneous surgical intervention. Given the benefits of reduced postoperative pain, enhanced aesthetic outcomes, and shorter hospitalization periods, we have employed this technique in our patient. Andrea Amabile and colleagues achieved a favorable outcome by utilizing a robotic-assisted, totally endoscopic approach for the excision of a PFE located on the mitral valve [5]. Weilong Di and colleagues conducted a study documenting a case of PFE situated on the tricuspid annulus. The tumor was successfully excised using a beating heart technique; however, it should be noted that Weilong Di utilized a median sternotomy approach to access the tumor [8]. According to Weidong Li, the beating heart technique was employed to excise a tumor in a patient with concomitant coronary artery disease, with the aim of avoiding the use of cardioplegia and the related ischemic complications [8]. Given the favorable location of the tumor, we opted to employ a beating heart technique for the tumor resection; however, it is noteworthy that we utilized a non-robotic totally thoracoscopic approach to access the tumor.

Subsequent to the surgical intervention, both patients were extubated within a timeframe of 5 to 6 h and required a stay in the intensive care unit lasting between 12 and 24 h before being discharged 3 to 4 days post-operation. Our observations indicate that the utilization of a minimally invasive approach in managing PFE resulted in favorable outcomes for these two cases. In comparison to other authors, the length of hospitalization observed in our study is nearly equivalent [1, 13]. In our experience, we posit that the optimal therapeutic approach for treating PFE in specific patient subpopulations is minimally invasive surgery. In situations where this option is not readily accessible, we advocate for a prompt referral to a specialized medical center with the requisite proficiency and resources to perform a minimally invasive intervention.

The efficacy of a minimally invasive surgical approach in managing PFE has been consistently demonstrated throughout the article. In this study, we present two cases of PFE, one on the aortic valve and the other the tricuspid valve, which were successfully treated using a minimally invasive surgical approach, resulting in favorable outcomes.

## Data Availability

All of the material is available and owned by the authors and/or no permissions are required.

## Abbreviations

PFE: Papillary fibroelastomas
TTE: Transthoracic echocardiography
TEE: Transesophageal echocardiography
CCT: Cardiac computed tomography
CMR: Cardiac magnetic resonance
